# Wavelength Dependent Graphene Oxide-Based Optical Microfiber Sensor for Ammonia Gas

**DOI:** 10.3390/s21020556

**Published:** 2021-01-14

**Authors:** Saad Hayatu Girei, Mohammed Majeed Alkhabet, Yasmin Mustapha Kamil, Hong Ngee Lim, Mohd Adzir Mahdi, Mohd Hanif Yaacob

**Affiliations:** 1 Wireless and Photonics Networks Research Centre, University Putra Malaysia, Serdang 43400, Selangor, Malaysia; gireisaad3@gmail.com (S.H.G.); mohammed.alkhabet@gmail.com (M.M.A.); yasminmustaphakamil@gmail.com (Y.M.K.); mam@upm.edu.my (M.A.M.); 2 Department of Computer Engineering, Federal Polytechnic Mubi, Mubi 650113, Adamawa State, Nigeria; 3 Department of Chemistry, Faculty of Science, University Putra Malaysia, Serdang 43400, Selangor, Malaysia; hongngee@upm.edu.my

**Keywords:** optical fiber sensor, ammonia, graphene oxide, absorbance

## Abstract

Ammonia detection in ambient air is critical, given its implication on the environment and human health. In this work, an optical fiber tapered to a 20 µm diameter and coated with graphene oxide was developed for absorbance response monitoring of ammonia at visible (500–700 nm) and near-infrared wavelength regions (700–900 nm). The morphology, surface characteristics, and chemical composition of the graphene oxide samples were confirmed by a field emission scanning electron microscope, an atomic force microscope, X-ray diffraction, and an energy dispersion X-ray. The sensing performance of the graphene oxide-coated optical microfiber sensor towards ammonia at room temperature revealed better absorbance response at the near-infrared wavelength region compared to the visible region. The sensitivity, response and recovery times at the near-infrared wavelength region were 61.78 AU/%, 385 s, and 288 s, respectively. The sensitivity, response and recovery times at the visible wavelength region were 26.99 AU/%, 497 s, and 192 s, respectively. The selectivity of the sensor towards ammonia was affirmed with no response towards other gases.

## 1. Introduction

Ammonia (NH_3_) has emerged as an important building block in the manufacturing of many products we use daily, including plastics, textiles, dyes, and household cleaning solutions. It is also a nitrogen source for plant growth, which makes it an essential ingredient in fertilizers. Furthermore, it has been widely used as a preservative in the agriculture industry, nitrogen source in the beverage industry, curing agent in the leather industry, and an anti-corrosive in the petroleum industry [1,2]. However, a high concentration of NH_3_ constitutes a threat to the human body, and therefore its detection is vital. NH_3_ concentration of 500 ppm can cause immediate and severe irritation to the nose and throat, while a higher concentration of 1000 ppm or more can cause pulmonary edema, accumulation of fluid in the lungs, and even death [3]. Therefore, it has become imperative to develop sensors that can detect NH_3_ for environmental and industrial safety. The traditional electrical sensors with metal oxides are highly sensitive and well established. However, these sensors exhibit improved sensitivity only at high operating temperatures, and are not immune to the interference of radio frequencies [4]. In recent years, there has been increasing interest in optical fiber sensors due to their unparalleled advantages over their electrical equivalents, such as simplicity of operation, real-time monitoring, immunity of various sources of interference, e.g., radiofrequency activity, electromagnetic interference, and explosive environment [5,6]. These characteristics make them cost-effective, flexible, and inert for gas sensing applications. To date, a range of optical fiber sensors with high efficiency for NH_3_ gas detection have been reported. As an example, Cao et al. [7] reported NH_3_ gas sensing by a U-shaped plastic-clad silica optical fiber covered with bromocresol. Results revealed that a very fast response time of 10 s was obtained at 55.5 ℃ with air or argon as a carrier gas. Mishra et al. [8] employed an unclad optical fiber sensor coated with indium tin oxide and bromocresol nanocomposite for NH_3_ gas detection. The sensing performance, which was evaluated using the surface plasmon resonance sensing technique, reveals a sensitivity of 1.891 nm/ppm. In another study, Wang et al. [9] recently utilized a tapered microfiber interferometer structure coated with tungsten trioxide for NH_3_ gas sensing. The microfiber structure was fabricated by heating and pulling method. The experimental results showed that the prepared sensor has a high sensitivity towards NH_3_ gas.

Among the various types of optical fiber sensors, tapered optical microfiber sensors have continued to receive considerable attention, given their additional advantages of high sensitivity and miniature size [10]. The fabrication of a tapered optical microfiber is performed by simultaneously heating and pulling a section of an optical fiber into microscale diameter [11]. A tapered optical microfiber consists of a region with uniform and reduced diameter (the waist), bounded by a conical part, where the diameter varies to merge the tapered section with the unperturbed section of the optical fiber [12]. This allows access to the evanescent field of modes propagating through the tapered region. The access to the evanescent waves facilitates interaction with the surrounding medium. To measure a chemical entity, material sensitive to the parameter to be detected has to be deposited on the tapered region of the optical microfiber. The evanescent waves change with changes in the refractive index (RI) of a sensing material when exposed to a chemical under test [12,13]. Tapered optical microfiber sensors have been used substantively in many sensing applications, ranging from environmental monitoring to healthcare. For instance, tapered optical microfiber-based sensors are used for temperature [14] and humidity [15] monitoring, to detect chemicals such as NH_3_ gas [16], oil and noxious spills in seawater [17].

In recent years, various kinds of sensing material, including metal oxide, carbon nanotube, graphene, and its derivatives have emerged. In particular, graphene oxide (GO) is considered an excellent material for gas sensing due to its high electron mobility, low electrical noise, and large surface area. GO also contains different oxygen functional groups such as carboxyl, hydroxyl, carbonyl, and epoxide with high sensitivity to surface adsorbate [18]. Previous studies have corroborated the use of GO as an attractive material for fabricating highly sensitive chemical sensors. Yu et al. [19] and Shabaneh et al. [20] developed GO-coated tapered optical microfiber sensors for the detection of NH_3_ and ethanol, respectively. The sensors demonstrated better sensing performance compared to the uncoated tapered optical microfiber. Sensitivities of 0.00035 nm/ppm and 0.0275 AU/% for NH_3_ and ethanol were obtained, respectively, which can be ascribed to the changes of the RI of GO when NH_3_ molecules are adsorbed on the surface of GO. Visible light utilization is becoming increasingly popular for communication and sensing applications such as visible light identifiers hospital robots, and in-vehicle network applications [21]. In [22], a visible light RGB demultiplexer was design based on multi-core polymer optical fiber, thus providing an opportunity for integrating optical fiber sensors based on visible light into the optical fiber telecommunication network. 

In this paper, an optical sensor for NH_3_ gas is presented using a GO-coated tapered optical microfiber. We have studied the absorbance characteristics of GO when introduced to different concentrations of NH_3_ gas (0.04–0.5%) and discovered that the sensitivity of the sensor has a significant dependency on the operational wavelength region (bandwidth). The sensor exhibited negative absorbance change in the visible wavelength region of 500–700 nm when exposed to an increasing amount of NH_3_. On the contrary, positive absorbance changes were observed in the near-infrared wavelength region of 700–900 nm when the sensor was exposed to the same concentrations of NH_3_ gas.

## 2. Experimental Details

### 2.1. Sensor Fabrication

**Figure 1:**
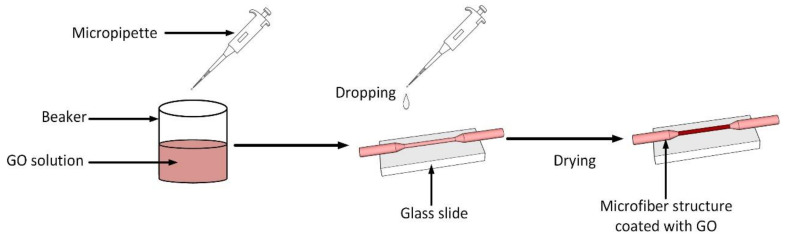
Schematic diagram for graphene oxide (GO) coating procedure onto optical microfiber.

The fabrication of the tapered optical microfiber was performed using the Vytran glass processing machine according to the procedure reported in [10]. Briefly, at first, the polymeric coating of the optical fiber was stripped for several centimeters using a fiber stripper and then washed with alcohol. The optical fiber was then mounted on the glass processing machine, where the area to be tapered is just above the filament. The two ends of the optical fiber were attached to the fiber holding stage. The bare multimode silica optical fiber, with core and cladding diameters of 62.5 and 125 µm, was then softened by the filament as a heating element. Once softened, the optical fiber was stretched by the holding stage at a constant pulling speed of 1 mm/s with filament heating power set at 38 W to attain a waist length of 15 mm and waist diameter of 20 µm. Next, the sensor was fixed on a glass substrate, washed with ethanol and deionized water to remove impurities, and subsequently placed in an oven with a temperature of 70 °C for 0.5 h. We used GO powders that were synthesized using the simplified Hummer’s method [23]. Later, 50 mg of as-prepared GO sample was dispersed in 50 mL of deionized water and sonicated for 1 h. A drop of GO solution (30 µL) was cast onto the tapered region. The whole structure was placed in an oven with a temperature of 40 °C for 4 h. Figure 1 shows the schematic for the preparation of GO coating on the tapered optical microfiber sensor.

### 2.2. Material Characterization

The morphology and molecular composition of GO were investigated using a field scanning electron microscope (FESEM, FEI Nova Nano-SEM 400) and energy dispersion X-Ray (EDX) spectroscope. The surface topography analysis was carried out using an atomic force microscope (AFM, NT-MDT Solver NEXT). The crystal phase was studied using an X-ray diffractometer (XRD, Bruker D8) with Cu Kα radiation at 1.5406 Å.

### 2.3. Experimental Setup

**Figure 2:**
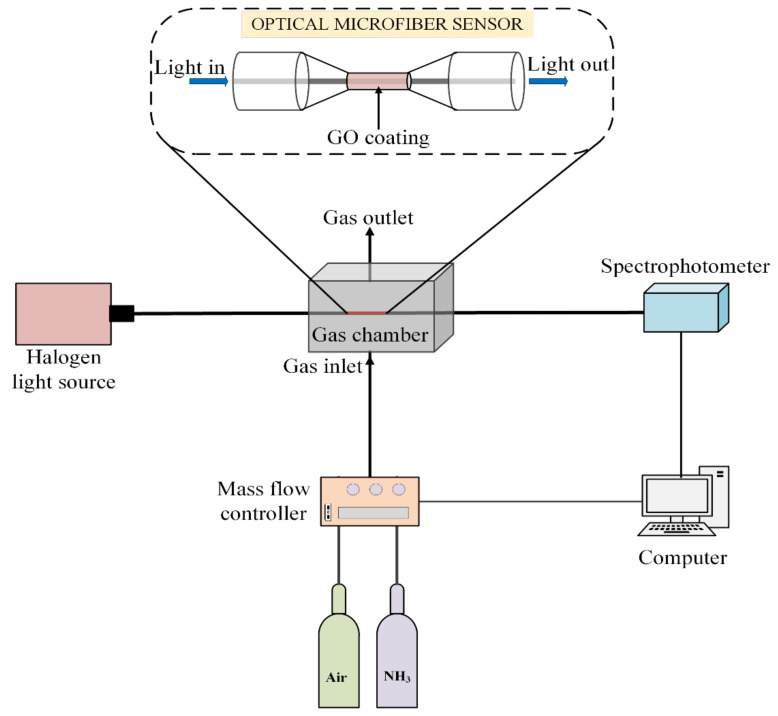
Schematic of the experimental setup for optical microfiber ammonia (NH_3_) gas sensor.

The experimental setup for the detection of the NH_3_ gas is shown in Figure 2. The sensing performance was characterized using a white light halogen source (Ocean Optics, HL-2000, Dunedin, FL, USA) of wavelength 360–2400 nm, and a portable spectrophotometer (Ocean Optics, USB 4000, Dunedin, FL, USA) with a wavelength range of 200–1100 nm to measure the absorbance. The serial dilution of NH_3_ in synthetic air into the sensing chamber was performed by a mass flow controller that regulates gas flow at 200 sccm. The concentration of NH_3_ in the chamber was varied by diluting 0.50% NH_3_ gas with high purity synthetic air. Absorbance measurements were performed while the sensor was exposed to NH_3_ with concentrations ranging from 0.04 to 0.50%. The absorbance spectrums were recorded using SpetraSuite software from a computer. The absorbance changes $\left(A_{\lambda }\right)$, corresponding to different concentrations of NH_3_ were calculated using the following equation:

(1) $$A_{\lambda }=-\log_{10}\left(\frac{S_{\lambda }-D_{\lambda }}{R_{\lambda }-D_{\lambda }}\right)$$

where $S_{\lambda }$ is the detected light intensity at wavelength λ when ammonia gas is present, $D_{\lambda }$ is the intensity when no light is injected into the optical fiber, and $R_{\lambda }$ is the reference intensity when synthetic air is present. The sensing performance was monitored at visible and near-infrared wavelength regions of the spectrum for different concentrations of NH_3_. The work was carried out under normal atmospheric pressure and room temperature (25–26 °C).

## 3. Results and Discussion

### 3.1. Characterization of Graphene Oxide

**Figure 3:**
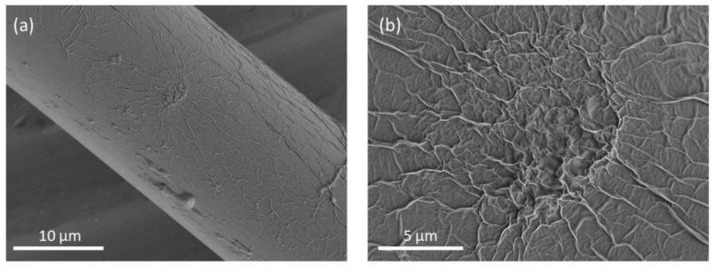
(**a**) Field scanning electron microscope (FESEM) image of GO-coated optical microfiber; (**b**) magnified image of the GO surface.

**Figure 4:**
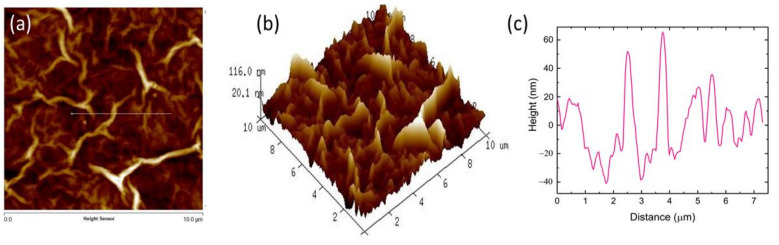
(**a**) 2D; (**b**) 3D topography of the atomic force microscope (AFM) image of GO film; (**c**) topographic curve of the GO film.

From the FESEM image of GO-coated tapered optical microfiber as shown in Figure 3a, it can be observed that the GO nanomaterial adhered well to the surface of the optical fiber. Figure 3b shows a magnified FESEM image of the GO-coated optical microfiber exhibiting a corrugated and wrinkled structure, which can be linked to the edges of the GO sheets [24]. The wrinkling is significant for GO surfaces in providing a high surface area for strong sensing performance. Figure 4a,b depict the two and three-dimensional AFM topography of the GO thin film, respectively, with a scan boundary area of 10 μm × 10 µm. The average surface roughness of the GO film was found to be ~24.12 nm. Rough sensing surface is important because it facilitates the diffusion of gas molecules into or out of the sensing layer [25]. Figure 4c shows the topographic curve of GO, which reveals an average height difference between rows of ~67.10 nm.

**Figure 5:**
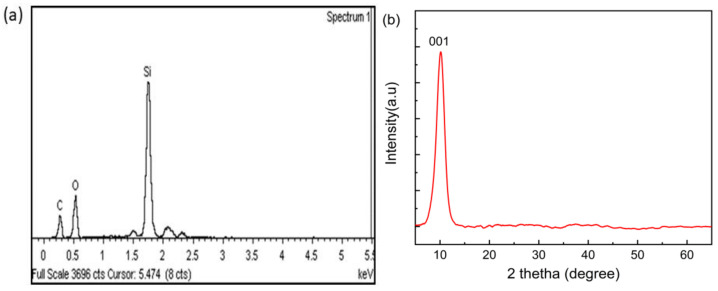
(**a**) Energy dispersion X-ray (EDX) spectrum of the GO-coated optical microfibers; (**b**) X-ray diffractometer (XRD) pattern of the prepared GO.

The EDX analysis shown in Figure 5a confirms the attachment of GO on the surface of the tapered optical fiber with the presence of C and O. The Si peak is due to the silica (SiO_2_) material of the optical fiber. Figure 5b shows the X-ray diffraction pattern of the GO sheet. The diffraction carbon peak for GO at 2θ = 10.13° on reflection plane (001) corresponds to an interlayer spacing of about 0.87 nm, confirming the presence of oxygen-containing functional groups [23].

### 3.2. Gas Sensing Performance

**Figure 6:**
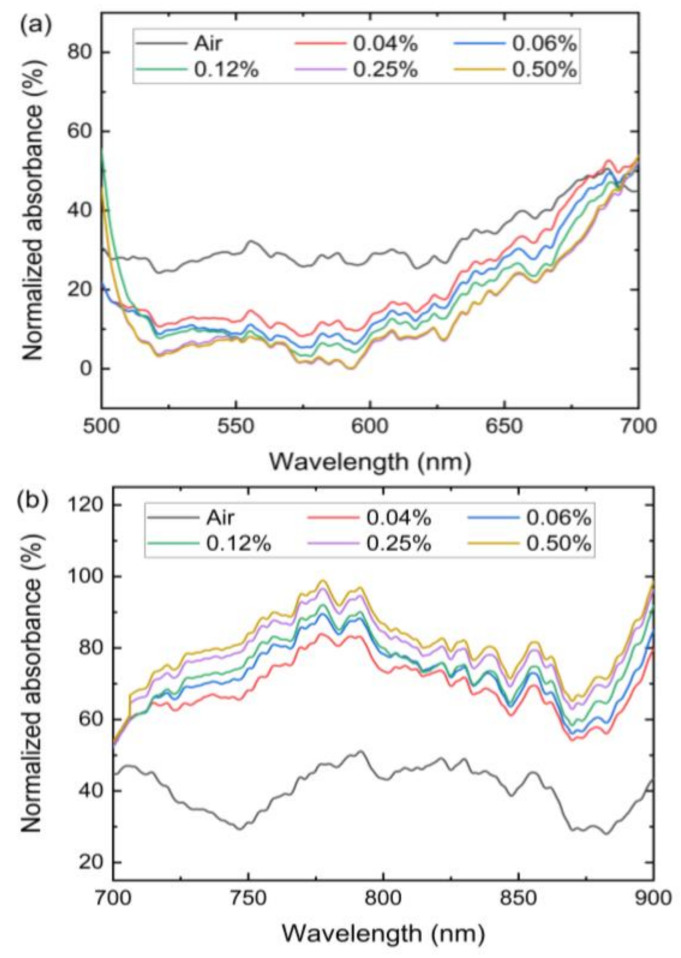
Absorbance spectra for GO-coated optical microfiber sensor in (**a**) visible wavelength region (**b**) near-infrared wavelength region under varying NH_3_ gas concentrations.

Figure 6a shows the absorbance behavior of the spectrum 500–700 nm when exposed to different concentrations of NH_3_. It can be observed that the absorbance response in this wavelength range changes with different concentrations of NH_3_. However, the nature of the absorbance change is different in the near-infrared wavelength range of 700–900 nm, as can be observed in Figure 6b. It can be noted that the absorbance in the visible wavelength range decreased with an increase in NH_3_ concentration while the absorbance in the near-infrared wavelength range increased with an increase in NH_3_ concentration. The absorbance response depends on the RI of the GO upon exposure to targeted gas, which in this case is NH_3_. The negative response of absorbance at the visible wavelength region indicated that the output power at the end of the sensor increased when the NH_3_ was exposed to the sensor. Therefore, at the visible wavelength region, the GO nanomaterial yielded low or negative absorption when exposed to NH_3_ gas. The opposite was the case when the sensor was exposed to NH_3_ gas in the near-infrared wavelength region.

**Figure 7:**
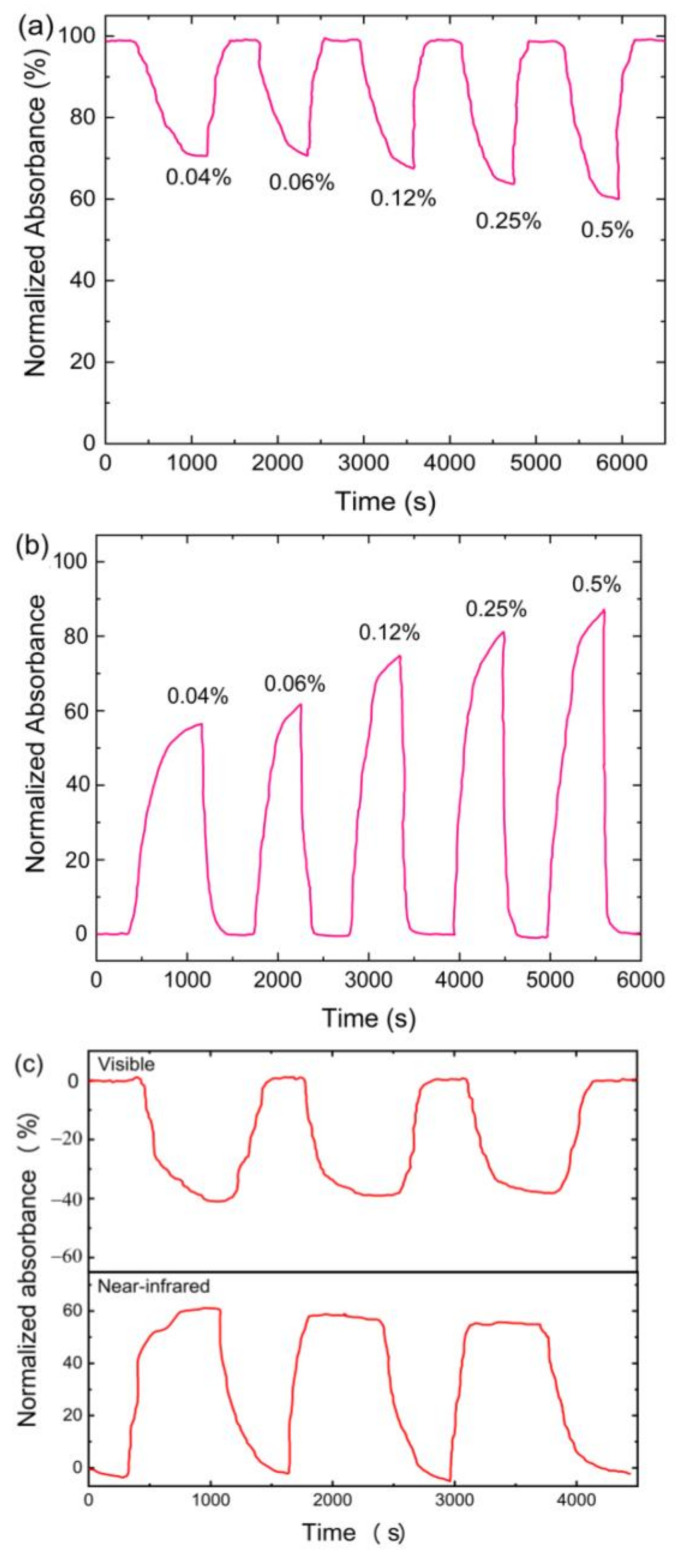
Dynamic response of the GO-coated microfiber sensor at (**a**) visible wavelength (500–700 nm); (**b**) near-infrared wavelength (700–900 nm) under varying NH_3_ gas concentrations; (**c**) repeatability of the GO-coated optical microfiber sensors exposed to 3 cycles of 0.04% NH_3_.

Figure 7a,b shows the dynamic response of the sensor when exposed to different NH_3_ concentrations at the visible and near-infrared wavelength regions, respectively. When the sensor was exposed to synthetic air, the absorbance dropped to its initial value, indicating a stable baseline and good reversibility of the GO-coated sensor. From Figure 7a the response of the sensor decreased linearly with an increase in NH_3_ gas concentration within the visible wavelength range. Absorbance response of 26, 28, 31, 35 and 39% were obtained for 0.04, 0.06, 0.12, 0.25 and 0.50% concentration of NH_3_ gas, respectively. For the absorbance response at near-infrared wavelength region, magnitudes of 58, 60, 72, 81, and 88 were obtained for 0.04, 0.06, 0.12, 0.25, and 0.50% concentration of NH_3_ gas, respectively. This observation is attributed to the charge transfer between NH_3_ molecules and GO sheet, which led to a change in RI of GO. Response and recovery time are crucial parameters of consideration in gas sensing applications. For 0.04% NH_3_ gas, the response and recovery at visible wavelength range were found to be 479 s and 192 s, respectively. Likewise, for the near-infrared wavelength range, the response and recovery time was found to be 385 s and 288 s, respectively. The fast recovery of the GO-coated sensor to its base absorbance indicates that NH_3_ molecules desorb faster from the GO surface. The repeatability of the GO-coated sensor at the visible and near-infrared wavelength region is shown in Figure 7c, which were obtained by exposing the sensor to 0.04% NH_3_ for three cycles at room temperature. After repeated exposure to NH_3_ gas, it can be observed that the absorbance of the sensor for both wavelength regions was maintained. It can be observed that the fabricated sensor at both wavelength regions showed good stability and repeatability, which is critical for accurate gas detection and practical application.

**Figure 8:**
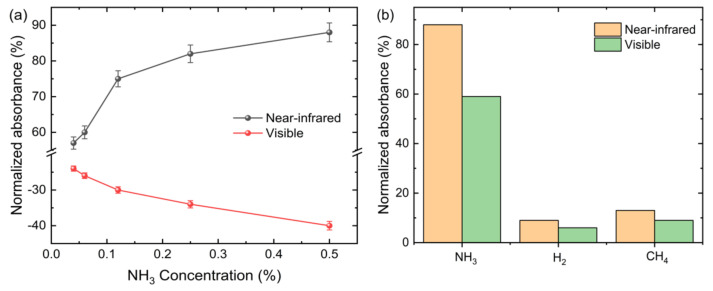
(**a**) Absorbance response changes as a function of NH_3_ concentrations for visible and near-infrared wavelength range; (**b**) selectivity of the optical microfiber sensor against H_2_, and CH_4_.

The plot of absorbance change against NH_3_ concentration is shown in Figure 8a. The changes in the absorbance depend on the NH_3_ concentration. In our experiment, the Sensitivity S is defined as the slope of the calibration graph given by S = ΔA/ΔC, where ΔA is the change in absorbance response, and ΔC is the change in NH_3_ gas concentrations. The near-infrared wavelength region showed higher sensitivity towards NH_3_ gas concentration compared to the sensitivity observed in the visible wavelength region. The sensitivities of 26.99 AU/% and 61.78 AU/% were obtained for the visible and near-infrared wavelength region, respectively. The sensitivity in the near-infrared wavelength region was more than twice the sensitivity in the visible wavelength region. Thus, operational bandwidth plays a role in determining the sensor’s sensitivity. The RI variation of GO film in response to NH_3_ gas exposure depends on factors such as film thickness, absorption properties, and wavelength of light. Previous studies indicated that the sensitivity of NH_3_ gas to materials such as GO [26] and polyalanine [27] depends also on the wavelength of light used. Suppose that GO film has a complex RI of η_c_ = η_r_ + ik where η_r_ is the real part of the RI and k is the extinction coefficient. The extinction coefficient is related to the absorption coefficient, α = 4πk⁄λ where λ is the wavelength of light. The RI of material corresponds to a change in the propagation of light due to dielectric interactions and varies with the wavelength [26]. The easy transfer of GO to the microfiber by drying GO solution and provision of high surface area by the corrugated and wrinkled structure of GO enabled larger surface interaction between the GO and NH_3_ molecules at room temperature. The GO response towards NH_3_ gas reveals negative and positive absorbance responses for the visible and near-infrared wavelength regions, respectively. Therefore, this work provides an insight into the wavelength region where the sensitivity of the sensor is the highest. 

The theoretical limit of detection (LOD) of the GO-coated sensor was calculated to be 21 and 13 ppm in visible and near-infrared wavelength regions, respectively. The LOD was derived from Figure 6a using the following equation [28].

(2) $$LOD=\frac{S_{a}}{b}$$

where $S_{a}$ is the standard deviation of the response and *b* is the slope of the calibration curve in Figure 8a, the LOD of the GO-coated tapered optical fiber sensor is below the maximum allowable level of 35 ppm recommended by the occupational safety and health administration (OSHA). The LOD of the developed sensor is compared to the previous studies as shown in Table 1.

Selectivity is an important key to consider in measuring gas sensing properties. The absorbance characteristics of the sensor towards H_2_ and CH_4_ gases were examined, and the results are shown in Figure 8b. The figure indicates that the GO-coated sensor exhibits good selectivity to NH_3_ at room temperature. The response of the sensor to 0.5% NH_3_ in the near-infrared wavelength region is eight times higher than the other gases. This is due to the larger absorption and stronger charge transfer capability of NH_3_, attributed to the surface epoxy or hydroxyl groups on the basal plane of GO that promotes adsorption of NH_3_ [32]. Furthermore, the adsorption nature of graphene-based materials is highly selective to polar molecules such as NH_3_ and has much lower sensitivity towards nonpolar molecules such as H_2_ and CH_4_ [33].

### 3.3. Sensing Mechanism

**Figure 9:**
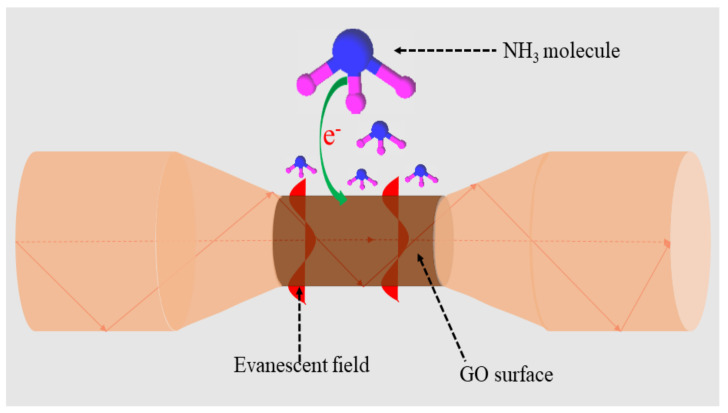
NH_3_ gas sensing mechanism of GO-coated optical microfiber sensor.

Figure 9 illustrates the proposed sensing mechanism of the sensor. The sensing mechanism of the GO-coated optical microfiber sensor at room temperature can be explained based on charge transfer between NH_3_ and GO. When NH_3_ is adsorbed by GO, it donates an electron on the surface of the carbon [34] as indicated in Equation (3). This charge transfer process would affect hole concentration in the GO, thereby changing its RI accordingly.

(3) $$NH_{3}\to NH_{3}^{\delta +}+e^{-}$$

The formation of vacancy defects, such as dangling bonds, on the GO surface provides higher absorption capacity and improves sensitivity. Additionally, surface-active defect sites such as epoxy and hydroxyl groups of GO promote interactions of NH_3_ molecules with GO film [19,32]. In the present study, the GO-coated optical microfiber sensor showed excellent sensing performance in both visible and near-infrared wavelength regions.

## 4. Conclusions

In conclusion, a room temperature NH_3_ sensor based on tapered optical microfiber coated with GO was demonstrated. The experimental result of the GO-coated sensor showed that the RI of the GO was sensitive to varied concentrations of NH_3_ at different wavelength regions. The sensor responds to the changes in NH_3_ concentrations by achieving a sensitivity of 26.99 AU/% and 61.78 AU/% in visible and near-infrared wavelength regions, respectively. It also showed strong recoverability and repeatability with a LOD of 13 ppm in the near-infrared wavelength region. The fabricated sensor showed highly distinguished selectivity towards NH_3_ in comparison with H_2_ and CH_4_ gases. The developed sensor has good prospects and may find applications in industrial, health, and agricultural fields, for instance, in the manufacture of fertilizers, textiles, plastics, and for environmental monitoring. 

## Figures and Tables

**Table 1:** Comparison of the developed sensor and other related optical fiber sensors.

Sensor Structure	Materials	LOD (ppm)	Ref.
Unclad optical fiber	PMMA/rGO	10	[29]
Polymer clad silica optical fiber	Reagent	31	[30]
Side-polished optical fiber	GO/PANI	22.46	[25]
Etched tapered optical fiber	PANI	25	[31]
Tapered optical fiber	GO	13	This work

## Data Availability

Not applicable.
